# Portable Measurement System for the Characterization of Capacitive Field-Effect Sensors

**DOI:** 10.3390/s25092681

**Published:** 2025-04-24

**Authors:** Tobias Karschuck, Stefan Schmidt, Stefan Achtsnicht, Joey Ser, Ismail Bouarich, Georges Aboutass, Arshak Poghossian, Patrick H. Wagner, Michael J. Schöning

**Affiliations:** 1 Institute of Nano- and Biotechnologies, FH Aachen, 52428 Jülich, Germany; 2 Laboratory for Soft Matter and Biophysics, KU Leuven, 3001 Leuven, Belgium; 3 Institute of Pharmaceutical Chemistry, Phillipps University of Marburg, 35037 Marburg, Germany; 4 MicroNanoBio, 40479 Düsseldorf, Germany; 5 Institute of Biological Information Processing (IBI-3), Forschungszentrum Jülich GmbH, 52425 Jülich, Germany

**Keywords:** field-effect capacitor, portable measurement system, pH sensor, penicillin biosensor

## Abstract

A user-friendly, portable, low-cost readout system for the on-site or point-of-care characterization of chemo- and biosensors based on an electrolyte–insulator–semiconductor capacitor (EISCAP) has been developed using a thumb-drive-sized commercial impedance analyzer. The system is controlled by a custom Python script and allows to characterize EISCAP sensors with different methods (impedance spectra, capacitance-voltage, and constant-capacitance modes), which are selected in a user interface. The performance of the portable readout system was evaluated by pH measurements and the detection of the antibiotic penicillin, hereby using EISCAPs consisting of Al/p-Si/SiO_2_/Ta_2_O_5_ structures and compared to the results obtained with a stationary commercial impedance analyzer. Both the portable and the commercial systems provide very similar results with almost perfectly overlapping recorded EISCAP signals. The new portable system can accelerate the transition of EISCAP sensors from research laboratories to commercial end-user devices.

## 1. Introduction

Semiconductor field-effect devices (FEDs) based on electrolyte–insulator–semiconductor (EIS) systems (e.g., ion-sensitive field-effect transistor (ISFET), silicon nanowire transistor, extended-gate FET) are widely recognized as an encouraging and generic device concept in the chemo- and biosensing field [1,2,3,4]. Owing to their device architecture and operating mechanism, solution-gated FEDs are, in principle, able to detect any charge alteration caused by biochemical reactions or biomolecular interactions at or near the gate surface. Recently developed device designs such as dielectrically-modulated nanogap FEDs are also able to detect neutral species [5,6]. FEDs offer remarkable benefits, such as miniature size, rapid response time, and their ability for label-free, real-time, and multiplexed detection of different analytes.

A field-effect EIS capacitor (EISCAP) is commonly accepted as the simplest FED and represents a chemically/biologically sensitive capacitor [7]. The operating principle of an EISCAP is based on altering the semiconductor space-charge (depletion) capacitance induced by the biochemical interactions at the gate surface. Thanks to the straightforward layout and the uncomplicated, cost-effective manufacturing, EISCAPs are applied widely for characterizing novel biochemically sensitive materials as well as for chemo- and biosensing applications. For example, EISCAPs functionalized with appropriate sensitive materials or receptors were employed for measuring pH [8,9,10] and ion concentrations [11,12], for detecting metabolites [13,14,15,16], antibiotics (e.g., ampicillin [17], penicillin [18,19,20]), small molecules and protein biomarkers [21,22,23,24,25,26], DNA-hybridization [27,28], charged nanoparticles/viruses [29,30,31,32,33] as well as for designing biomolecular logic gates, which mimic electronic logic gates [34]. Emerging application fields of EISCAP-based chemo- and biosensors reach from biotechnology, environmental monitoring, point-of-care, and personalized medicine up to food and drug safety purposes [7]. However, a reliable, accurate, miniaturized electronic readout system for such sensors is one of the main demands in this field.

The sensitive parameters of EISCAPs are usually characterized in the capacitance-voltage (*C*−*V*) and/or constant-capacitance (ConCap) measurement modes [7,35,36]. Additionally, gate-leakage current and impedance characteristics are often measured to prove the correct functioning of the EISCAP sensor and to determine the preferred frequency range for *C−V* and ConCap measurements. Typically, EISCAP sensors are electrochemically characterized by expensive and bulky stationary impedance analyzers, which are accurate and well-suited for solving scientific problems in laboratory settings. However, because they are not portable, their application potential is limited. Portable and easy-to-use readout systems to characterize EISCAP-based chemo- and biosensors can play a substantial role in their future commercialization. While many examples of self-made (see, e.g., [37,38,39,40,41,42,43]) or commercial [44,45,46,47] portable detection systems have been reported for ISFET-based chemo- and biosensors, there are only a few publications on easy-to-use, stand-alone portable systems for characterizing EISCAP sensors. For example, a homemade lab-type impedance analyzer for EISCAPs was reported in [48] as a user-friendly device, allowing to operate EISCAP sensors for on-site pH measurements in cell-culture media. However, due to limitations regarding the AC (alternating current) frequency range (125–100 kHz), a narrow capacitance measuring range (2–60 nF), and the lack of a light-excluding box to ensure reproducible EISCAP measurements outside a Faraday cage, this portable device has not found wide application. Veeramani et al. reported on a portable readout system based on an unconventional frequency-mode measurement by putting the EISCAP in an oscillator loop, whose frequency is modulated by pH changes of the solution [49,50]. Drawbacks of this portable system include the use of an unstable “liquid-free”, pseudo-reference electrode and a complex calibration procedure.

Recently, portable impedance analyzers have become readily available at low cost, mainly driven by the popularity of impedimetric biosensors (see, e.g., [51,52,53,54,55,56]). Using a market-ready solution from an original equipment manufacturer (OEM) may offer advantages such as production scalability, easy supply with components, warranty, and support during certification procedures. While the impedance spectra and *C−V* curves can generally be recorded by many portable commercial impedance analyzers, time-resolved ConCap mode readout of the EISCAP signal is typically not a standard feature. To commercialize EISCAP-type chemo- and biosensors for on-site and point-of-care applications, the readout hardware must be readily available, portable, inexpensive, allow for facile data exchange (e.g., USB interfaces), and user-friendly software is needed. To allow non-specialized staff to operate EISCAP biosensors, the measurement routines (impedance spectra, *C−V-* and ConCap mode) should be selectable in a user interface and run automatically once a measurement is started.

In this work, we present an easy-to-use portable PC-controlled readout system based on the commercial potentiostat “Sensit Smart” from a leading developer (PalmSens, Houten, The Netherlands) [57]. Combined with a base station and a custom-made measuring cell that hosts the EISCAP chip, this system meets all requirements. The “Sensit Smart” was selected because it is one of the smallest and most affordable potentiostats offered by an OEM. The performance of the developed portable system, controlled by a custom Python script, is evaluated on two test cases: pH measurements and the detection of penicillin. All data are benchmarked with respect to a stationary, state-of-the-art impedance analyzer, “IM6ex” (Zahner-Elektrik, Kronach, Germany). To the best of our knowledge, this is the first attempt to use a commercially available USB-drive-sized impedance analyzer in a portable measurement system for characterizing EISCAP biosensors.

## 2. Concept of the Portable Measurement System for EISCAP Sensors

**Figure 1:**
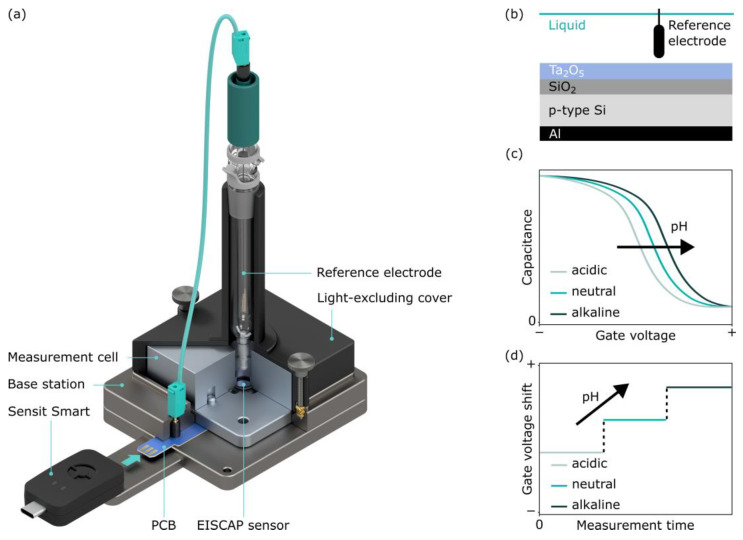
A render of the developed portable measurement system consisting of the commercial “Sensit Smart” (modified from [58]), an adapter base station, a measurement cell with the installed EISCAP chip, a reference electrode, and a light-excluding cover (**a**). Schematic structure of the pH-sensitive Al/p-Si/SiO_2_/Ta_2_O_5_ EISCAP sensor (**b**), typical shape of the pH-dependent responses in high-frequency *C−V* (**c**) and ConCap mode (**d**).

The developed portable measurement system consists of the “Sensit Smart”, an adapter base station that includes the assembled printed circuit board (PCB), the measurement cell with the installed EISCAP chip, a reference electrode (RE), and a light-excluding cover (see Figure 1). In the following sections, we will describe all components of the system as well as a custom-developed Python script used to control the “Sensit Smart”. For stand-alone operation, the portable measurement system is operated by the Python script running on a laptop, which is connected to the “Sensit Smart” via a USB interface.

### 2.1. Preparation and Functioning Principle of EISCAPs

Figure 1b illustrates a schematic structure of the pH-sensitive Al/p-Si/SiO_2_/Ta_2_O_5_ EISCAP sensor used in this study. The preparation of these sensors was described previously [19,32]. In summary, a commercially available p-Si wafer covered with a 30 nm SiO_2_ layer (Siegert Wafer, Aachen, Germany) was used to deposit a 60 nm Ta_2_O_5_ pH-sensitive layer by electron-beam evaporation of tantalum and subsequent oxidation in oxygen atmosphere at 520 °C for 60 min. After etching the backside of the wafer with hydrofluoric acid, a 300 nm Al layer was added by electron-beam evaporation as a contact layer to the p-Si.

The wafer was diced into 10 mm by 10 mm sensor chips, which were cleaned in acetone, isopropanol, ethanol, and distilled water for 3 min in an ultrasonic bath. Finally, the EISCAP chips were mounted in measurement cells and sealed with an O-ring to protect the Al contact and p-Si sidewalls from the solution.

The operating principle of a pH-sensitive EISCAP is based on the detection of charge changes on the gate-insulator (here, Ta_2_O_5_) surface induced via the protonation or deprotonation of surface groups [7,59,60]. This pH-dependent alteration of the surface charge will modulate the width of the space-charge region in the semiconductor and, thereby, the capacitance of the EISCAP. The variation of the capacitance of the EISCAP in response to pH results in a shift of the *C–V* curve along the voltage axis. For a p-type EISCAP studied in this work, a pH increase will shift the *C–V* curve toward more positive gate voltages, while a pH decrease will shift the *C–V* curves toward less positive (or more negative) gate voltages (see Figure 1c). The direction and amplitude of potential shifts can be determined directly by the time-resolved ConCap-mode response (Figure 1d). In the ConCap-mode measurement, the total capacitance of the EISCAP at the selected working point is kept constant by controlling the applied gate voltage in a negative feedback loop. Usually, the working point for the ConCap-mode measurement is selected within the quasi-linear part of the depletion region of the *C–V* curve (often at 60% of the maximum capacitance in the accumulation region of the *C−V* curve [9]). An advantage of the ConCap method is the possibility of recording the EISCAP signal in real-time.

### 2.2. Hardware Components

The “Sensit Smart” from PalmSens is a thumb-drive-sized impedance analyzer with dimensions of 43 × 25 × 11 mm^3^ (excluding the USB connector), which weighs only 10 g and can be controlled by a smartphone. The operation temperature range for the “Sensit Smart” is 0 °C to 40 °C. It is widely used in portable electrochemical sensor systems for the detection of various targets, including SARS-CoV-2 [61,62,63,64], luteinizing hormone [65] and the antibiotic amoxicillin [66]. Using a “Sensit Smart”-based portable system offers impedance measurements with an applied gate voltage range of −2 V to +1.2 V, an additional AC voltage of 1–250 mV, and a frequency range from 16 mHz to 200 kHz. The “Sensit Smart” fulfills the requirements for the electrochemical characterization of EISCAPs.

For a stand-alone portable setup, we designed an adapter base station that accommodates the measurement cell with an installed EISCAP chip and the RE. The base station has been designed to fulfill the following requirements: (1) nondestructive sensor installation and removal; (2) direct easy connection to the “Sensit Smart”; and (3) light exclusion (it is known that characteristics of FEDs may be influenced by ambient light [67,68]). Shielding of the EISCAP sensor from external light was accomplished with a light-excluding cover, which is secured in place with two thumbscrews. This also achieves the necessary mounting pressure for the spring-loaded contact pin, which is used to electrically connect to the EISCAPs’ backside. The RE is secured in place with a cylinder, which protrudes from the cover lid by using a standard 14/15 taper joint. A 2 mm banana plug in the base station enables the electrical connection of the RE, which closes the circuit. Making use of “Sensit Smart’s” push-in connection, a printed circuit board (PCB) with a thickness of 0.6 mm was designed to act as an electrical interface to the sensor and RE. The PCB connects the RE with the short-circuited reference- and counter-electrode terminals of the “Sensit Smart”, while the sensor is connected to the working electrode terminal.

### 2.3. Software Adaptation

As discussed in the Introduction section, the developed portable measurement system for EISCAP sensors should be able to work in various operation modes and generate corresponding plots, including impedance spectra, *C−V*, and ConCap curves. While the impedance spectra and *C−V* curves can be recorded by the “Sensit Smart” impedance analyzer, the dynamic ConCap mode readout of the EISCAP signal is not a standard feature. The portable device was powered and controlled by a laptop running a custom Python script (Python V. 3.11), which relies on PalmSens’ MethodSCRIPT (V. 1.3) for communication with the “Sensit Smart” [69]. The main programming challenges for the portable characterization of EISCAP sensors with the “Sensit Smart” are the implementation of impedance spectra, *C−V* and ConCap measurement modes in a custom graphical user interface.

**Figure 2:**
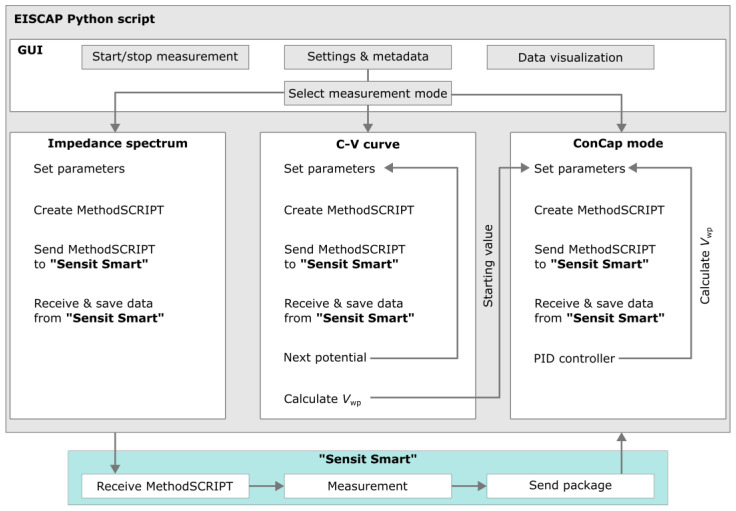
Block diagram of the EISCAP Python script with the measurement modes for impedance spectra, *C−V* curves, and ConCap characterizations using the “Sensit Smart”. PID (proportional-integral-derivative), *V*_wp_: working-point potential.

The main functions are displayed as a block diagram in Figure 2. The measurement routine from PalmSens for recording impedance spectra, using Python to send a MethodSCRIPT to the “Sensit Smart” and receive measurement data, was expanded to include the *C−V* and ConCap modes, respectively [70]. For *C−V* measurements, the MethodSCRIPT file was modified by the Python script to vary the gate voltage between –2 V and +1.2 V in adjustable increments with a set measurement frequency. The working-point capacitance *C*_wp_ can be selected either automatically by the script, at 60% of the maximum capacitance in the accumulation region of the *C−V* curve, or set manually in the graphical user interface. The corresponding working-point potential *V*_wp_ is automatically evaluated by the script by a linear fit between the two closest measured capacitances of the *C−V* curve to accurately determine *V*_wp_.

NumPy (V 1.24) was used for handling the measurement data [71]. We programmed the ConCap measurement as a new method for the “Sensit Smart” to characterize EISCAPs in a time-resolved manner. In ConCap mode, the simple-PID library (V 2.0) [72] was used to implement a feedback loop to control the required gate voltage and keep the selected working-point capacitance constant. An easy to navigate graphical user interface (GUI) for measurement-mode selection was built using CustomTkinter (V 5.2) [73]. Screenshots of the GUI can be found in the Supplementary Materials. Figures of the completed measurements were generated with matplotlib (V 3.7) [74] and can be viewed directly from the graphical user interface.

## 3. Results and Discussion

### 3.1. Comparative Study of EISCAPs in Impedance Spectroscopy, C−V, and ConCap Modes: Portable “Sensit Smart” vs. Stationary “IM6ex”

The performance of the developed portable measuring system based on the “Sensit Smart” was exemplarily evaluated by performing pH measurements and detecting the antibiotic penicillin. The “Sensit Smart” measurements were compared with results obtained by the state-of-the-art stationary impedance analyzer “IM6ex”. Therefore, the EISCAPs installed within the measurement cell were characterized in impedance spectroscopy, *C−V*, and ConCap modes. For the comparative study of EISCAPs between “Sensit Smart” and “IM6ex”, the measurement cell was placed in a dark Faraday cage.

#### 3.1.1. Measuring Conditions

Impedance spectra of the EISCAP pH sensors were recorded in Titrisol buffer solution (Merck, Darmstadt, Germany) of pH 7 at an applied gate voltage of −2 V (accumulation region) and in a frequency range varying between 20 Hz and 5 kHz. For *C*–*V*-mode measurements, a sweeping gate voltage between −2 V and +1 V with 100 mV steps and a superimposed AC voltage (for the capacitance measurement) with an amplitude of 20 mV and a frequency of 120 Hz were applied between the RE (Ag/AgCl double-junction reference electrode, Metrohm, Filderstadt, Germany) and the backside Al contact. The working point (i.e., the constant capacitance) for the ConCap-mode measurements was chosen from the recorded *C*–*V* curves. The *C*–*V* and ConCap plots were recorded in Titrisol buffers of pH 5–pH 9 (Merck, Darmstadt, Germany). Before the buffer exchange, the surface of the EISCAP chip was rinsed three times with the measurement buffer. The contact area of the EISCAP surface with the solution was 52 mm^2^. Before starting experiments, the EISCAPs were conditioned overnight in pH 7 buffer solution. Measurements were performed at room temperature in a buffer volume of 1 mL. All potential values are mentioned with respect to the Ag/AgCl electrode. Other experimental details are given in corresponding sections.

#### 3.1.2. Characterization of EISCAP pH Sensors by Means of Impedance Spectroscopy, C−V, and ConCap Modes

**Figure 3:**
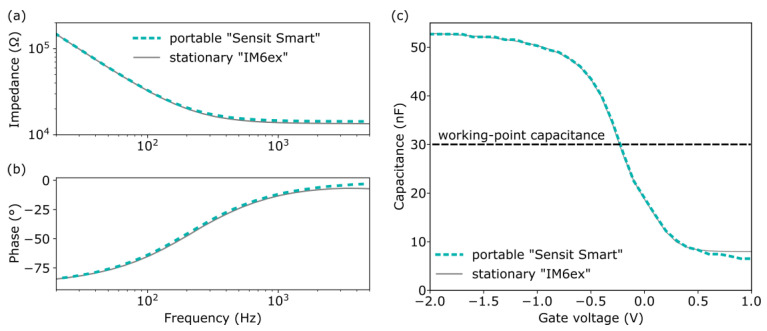
Comparison of impedance spectra (**a**), phase angles (**b**) of the EISCAP sensor in the accumulation region at a gate voltage of –2 V, and *C–V* curves (**c**) collected in Titrisol pH 7 buffer using the portable “Sensit Smart” and the stationary “IM6ex”.

Impedance spectra of the same EISCAP pH sensor were recorded in the accumulation region at −2 V by means of both the “Sensit Smart” and the “IM6ex”. As can be seen in Figure 3, both impedance plots are nearly identical. As expected, the measured impedance at low frequencies (f < 200 Hz) is dominated by the sensors’ capacitive reactance. With increasing frequency, the capacitive effect decreases as can also be seen by the decreasing phase angle, and a resistive plateau region became visible at frequencies from 1 kHz to 5 kHz. The impedance value in the plateau range, ca. 14 kΩ, can mainly be assigned to the resistance of the RE. The slight divergence between the phase plots recorded by the two devices at frequencies above 1 kHz can be attributed to the different cables used for the connection to the EISCAP chip. From the impedance spectra, a frequency of 120 Hz was selected for further characterization of EISCAPs in *C−V* and ConCap modes.

The high-frequency *C*–*V* plots measured with both the portable “Sensit Smart” and the stationary “IM6ex” are depicted in Figure 3c, where the characteristic regions of accumulation (−2 V to –1 V), depletion (–1 V to 0.5 V) and inversion (0.5 V to 1 V) can be distinguished. The recorded *C−V* curves of both devices are perfectly overlapping in the accumulation and depletion region. In the inversion region, starting at 0.5 V, the measured capacitances begin to diverge slightly by up to 2 nF. However, since EISCAPs typically operate in the depletion region, this small divergence in the inversion region has a negligible impact on the overall sensor performance.

**Figure 4:**
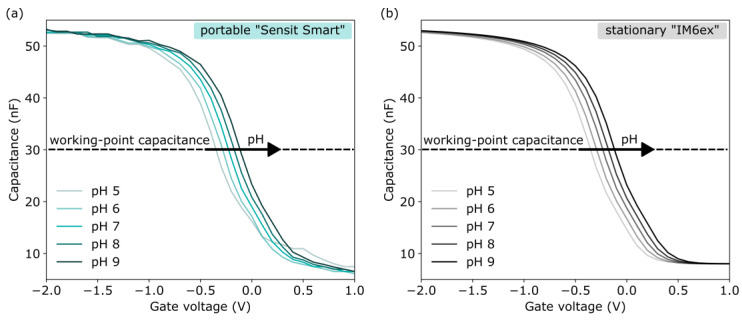
Set of *C−V* plots for an exemplary EISCAP pH sensor collected in Titrisol buffers from pH 5 to pH 9 using the portable “Sensit Smart” (**a**) and the stationary “IM6ex” (**b**).

After ensuring the correct operation, the EISCAPs were characterized in terms of pH sensitivity in Titrisol buffers with different pH values from pH 5 to pH 9. An exemplary set of the *C–V* plots of the EISCAP measured at different pH values, using both the portable device and the stationary “IM6ex”, is presented in Figure 4. As expected, with the increasing pH value of the buffer, the *C–V* plots are shifted along the voltage axis in the direction of less negative (or more positive) gate voltages, which corresponds to a more negatively charged gate surface. Shifts of the entire *C−V* curves along the voltage axis are clearly visible in the depletion region; the origin of these potential shifts has been discussed in Section 2.1. The EISCAPs’ pH-sensitivity values, evaluated from the depletion regions of the pH-dependent *C−V* plots in Figure 4, were practically identical with 59 ± 3 mV/pH (sensitivity ± standard error of the fit) for the “IM6ex” and 59 ± 2 mV/pH for the portable system. The working-point capacitance for the ConCap-mode measurements was selected in the depletion region at the inflection point of the *C−V* curve, as indicated by the dashed line at 30 nF.

**Figure 5:**
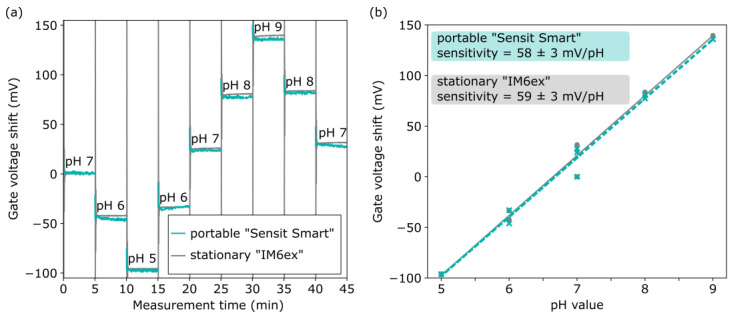
(**a**) ConCap responses of the pH-sensitive EISCAP recorded for pH values 7→6→5→6→7→8→9→8→7 using the portable “Sensit Smart” and the stationary “IM6ex”, (**b**) calibration plots of the EISCAP evaluated from the respective ConCap responses.

The ConCap responses of the pH-sensitive EISCAPs were recorded for pH values 7→6→5→6→7→8→9→8→7 using the portable measurement system and the stationary “IM6ex”. For a direct comparison of the results of these experiments, Figure 5a illustrates exemplary ConCap plots after offset correction (i.e., the sensor signals for the starting value at pH 7 were set to 0 mV) of the measured EISCAP signals. No datapoints were collected during the buffer exchange.

As can be seen, the ConCap plots, recorded with both systems, are almost perfectly overlapping. The pH-dependent signal shifts in the ConCap mode measurements are comparable to the shifts evaluated from the *C−V* plots. In both ConCap plots, certain drift and hysteresis phenomena are visible, independent of the used measurement device, and, therefore, most likely, may be attributed to the Ta_2_O_5_-gate EISCAP sensor. The calibration curves of the EISCAPs evaluated from the respective ConCap responses are provided in Figure 5b. The pH sensitivity of the EISCAP, calculated from the corresponding calibration curve, was in good agreement, namely, 59 ± 3 mV/pH and 58 ± 3 mV/pH by using the stationary “IM6ex” and the portable “Sensit Smart”, respectively. These results are consistent with the pH-sensitivity values reported for Ta_2_O_5_-gate field-effect sensors [9,10,75,76]. The near-perfect consistency in ConCap signal reproducibility after offset correction underlines the efficacy of the portable “Sensit Smart” in field-effect sensor applications.

#### 3.1.3. EISCAP-Based Penicillin Biosensor

In a further experiment, the performance of the portable “Sensit Smart” and the “IM6ex” impedance analyzer were compared by characterizing enzyme-modified EISCAP biosensors using penicillinase/penicillin as a model enzyme/substrate system. The EISCAP-based penicillin biosensor was created by adsorptive immobilization of the enzyme penicillinase (from *Bacillus cereus*, Merck, Darmstadt, Germany) on the Ta_2_O_5_-gate surface according to the procedure described in [20]. Briefly, 50 µL of 1 × PBS (phosphate-buffered saline, pH 7.4) containing dissolved penicillinase with an activity of 30 U was drop-coated atop the Ta_2_O_5_ layer of each individual EISCAP (overall, three EISCAP penicillin biosensors were prepared and tested) and incubated for 2 h at room temperature. After immobilization, the EISCAP surface was washed with measurement buffer (0.33 mM PBS, pH 7.4) to remove unbound penicillinase molecules and dried at room temperature.

The working principle of the penicillin-sensitive EISCAP biosensor is based on the detection of local changes of the H^+^-ion concentration near the gate surface, which is caused by the enzymatic hydrolysis of penicillin by penicillinase according to the following reaction:

(1) $$\mathrm{penicillin}\;\overset{\mathrm{penicillinase}}{\to }\mathrm{penicilloic}\;\mathrm{acid}+{\;H\;}^{+}$$

**Figure 6:**
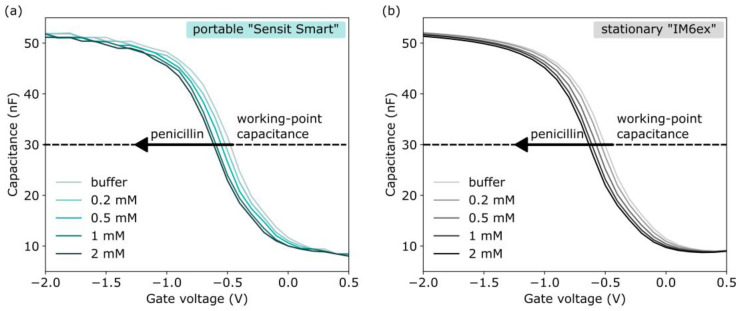
Set of *C−V* plots for the same EISCAP penicillin biosensor recorded in buffer and penicillin solutions with different concentrations from 0.2 mM to 2 mM using the portable “Sensit Smart” (**a**) and the stationary “IM6ex” (**b**).

The resulting penicillin-concentration-dependent pH decrease is detected by the underlying pH-sensitive EISCAP in the same way as described in Section 2.1 and Section 3.1.2. The EISCAP penicillin biosensors were characterized in *C−V*- and ConCap mode by starting with a baseline measurement in a 0.33 mM PBS buffer (pH 7.4, without penicillin), followed by measurements in the same buffer containing 0.2 mM, 0.5 mM, 1 mM, and 2 mM penicillin, respectively. Prior to starting the measurement, the sensors were rinsed three times with the measurement buffer. The high-frequency *C–V* curves of the EISCAP penicillin biosensor, recorded with the “Sensit Smart” and “IM6ex”, in penicillin solutions of different concentrations are shown in Figure 6. As expected, with increasing penicillin concentration from 0.2 mM to 2 mM, the *C–V* plots shift along the voltage axis in the direction, which corresponds to a pH decrease and a more protonated surface. From the *C−V* plots, the working-point capacitance for the ConCap measurements was set to 30 nF.

**Figure 7:**
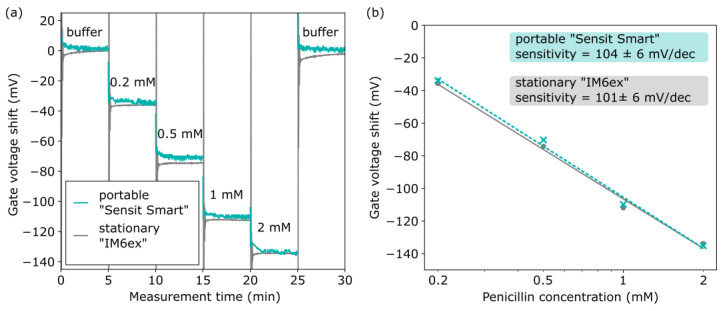
(**a**) ConCap responses of the same EISCAP penicillin biosensor measured in buffer and penicillin solutions with different concentrations from 0.2 mM to 2 mM using the portable “Sensit Smart” and the stationary device “IM6ex”; (**b**) calibration plots of the penicillin biosensor evaluated from the respective ConCap responses.

Figure 7 provides a comparison of the offset-corrected ConCap responses of the same EISCAP penicillin biosensor, measured with the portable “Sensit Smart” system and with the stationary “IM6ex” instrument for penicillin concentrations from 0.2 mM to 2 mM. For offset correction, the signals were first normalized in the penicillin-free buffer. As can be seen, the ConCap signals recorded with both measurement devices are almost identical. The penicillin sensitivities evaluated from the respective calibration curves in Figure 7b were 104 ± 6 mV/dec (sensitivity ± standard error of the fit) for the portable “Sensit Smart” system and 101 ± 6 mV/dec for the stationary “IM6ex”. The pH- and penicillin sensitivity values for three characterized EISCAP sensors display good reproducibility, as summarized in Table 1. The average penicillin sensitivity of the three sensors was evaluated at 102 ± 2 mV/dec (sensitivity ± standard deviation, *n* = 3) for the “Sensit Smart” and 98 ± 3 mV/dec for the “IM6ex”. The obtained average penicillin sensitivities are in the range of reported values for different types of enzyme-based FEDs [18,19,20,77,78].

### 3.2. pH Characterization of EISCAP with Portable Measurement System Outside of a Faraday Cage

**Figure 8:**
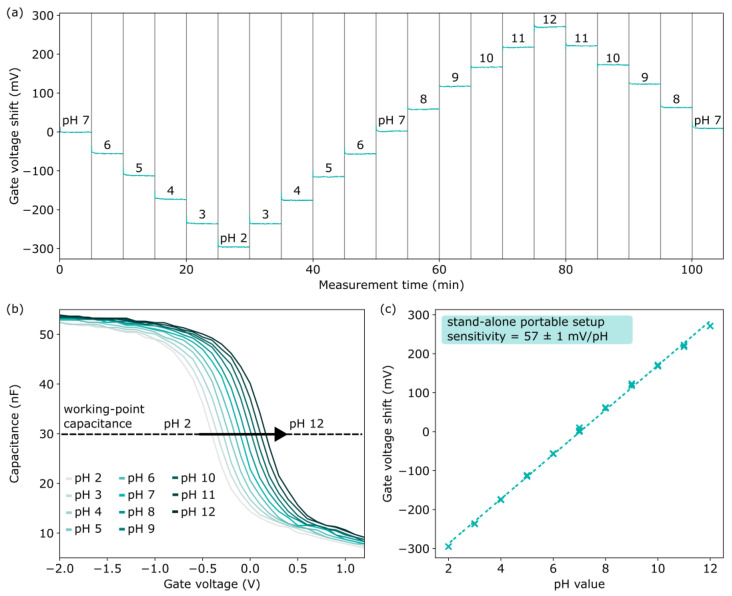
Exemplary ConCap response (**a**) and set of *C−V* plots (**b**) of a pH-sensitive EISCAP measured with the “Sensit Smart”-based portable measurement system in buffer solution with different pH values ranging from pH 2 to pH 12 outside of a Faraday cage; (**c**) calibration plot of the pH EISCAP evaluated from the ConCap measurement.

To show that the developed portable stand-alone measurement system (depicted in Figure 1a) is suitable for on-site applications, the subsequent experiments, see Figure 8, were performed outside of a Faraday cage in a laboratory environment under daylight.

Therefore, the pH-sensitive Ta_2_O_5_-gate EISCAP was characterized in a wide pH range from pH 2 to pH 12, with the chip installed in the measurement cell, which was mounted on the base station and enclosed by the light-excluding cover. The recorded sets of *C−V* plots and offset-corrected ConCap signals are illustrated in Figure 8a,b. These curves are very similar to those that had been measured before in the Faraday cage (see Figure 4 and Figure 5), verifying the effectiveness of the portable measurement system with its cover serving as a light-excluding box. Comparing *C−V* curves of this sensor for repeated measurements at pH 7 displayed a good reproducibility of the measured capacitance, with a standard deviation up to 0.53 nF. The Ta_2_O_5_-gate EISCAP possessed a nearly-Nernstian ConCap pH sensitivity of 57 ± 1 mV/pH (sensitivity ± standard error of the fit) over a wide pH range of pH 2–pH 12 (see Figure 8c).

## 4. Conclusions and Outlook

Portable, inexpensive, and easy-to-use readout systems for characterization of EISCAPs are expected to play a key role in commercializing EISCAP-based chemo- and biosensors for on-site and point-of-care applications in the future. In this study, we developed an easy-to-use portable, PC-controlled readout system consisting of a USB-drive sized, commercially available, and affordable “Sensit Smart” impedance analyzer (PalmSens, Houten, The Netherlands), an adapter base station, and a measurement cell with the mounted EISCAP chip, RE and a light-excluding cover plate. The system is controlled by a custom Python script, enabling the routine characterization of EISCAP sensors in different modes (impedance spectra, *C−V*, and ConCap), which are selectable in a user-friendly interface and run automatically once a measurement is started. The performance of the developed portable system was exemplarily evaluated for the detection of pH and the antibiotic penicillin, using EISCAPs consisting of an Al/p-Si/SiO_2_/Ta_2_O_5_ structure, and compared with the results obtained with a commercial stationary impedance analyzer “IM6ex” (Zahner-Elektrik, Kronach, Germany).

Impedance spectra and capacitance-voltage plots of the pH-sensitive EISCAPs measured with both systems at pH 7 were found to be almost perfectly overlapping. Close to Nernstian pH sensitivities of 58 ± 3 mV/pH (sensitivity ± standard error of the fit) and 59 ± 3 mV/pH were retrieved from the constant-capacitance plots of an EISCAP using the portable device and, respectively, the stationary impedance analyzer. These pH sensitivities are perfectly in line with the sensitivity of other Ta_2_O_5_-gate field-effect pH sensors.

To prove the performance of the portable system for the characterization of EISCAP biosensors, the enzyme penicillinase was successfully immobilized onto the Ta_2_O_5_ gate surface. The high average penicillin sensitivity of 102 ± 2 mV/dec (sensitivity ± standard deviation, *n* = 3) in the penicillin concentration range between 0.2 mM and 2 mM, as determined with the portable “Sensit Smart”, is comparable with the sensitivity of 98 ± 3 mV/dec obtained with the stationary “IM6ex” using the same EISCAP biosensors.

Additional experiments, which were performed outside of a Faraday cage (under daylight conditions) in a wide pH range of pH 2–pH 12, have verified the effectiveness of using a measurement cell in a base station with a cover serving as light-excluding box, hence demonstrating the suitability of the developed portable measurement system for on-site or in-field applications. Even in such a wide pH range, the studied Ta_2_O_5_-gate EISCAP displayed a nearly linear calibration curve and a high sensitivity of 57 ± 1 mV/pH (sensitivity ± standard error of the fit).

In the future, the developed portable system might be extended for the label-free detection of biomolecules and nanoparticles/viruses by their intrinsic charge. Moreover, the measurement cell will be constructed to provide a characterization of EISCAPs with different chip sizes. Although a laptop was used to power and control the portable system in the presented experiments, the setup can be easily adapted for applications using smartphones or tablets. We believe that the developed portable system can help accelerate the transition of EISCAP sensors from research laboratories to be applicable as commercial end-user devices.

## Figures and Tables

**Table 1:** Comparison of penicillin sensitivity for EISCAPs characterized by the portable device “Sensit Smart” and stationary impedance analyzer “IM6ex”. The sensitivity of individual sensors is given with the corresponding standard error of the fit. The average sensitivity of the three sensors is listed with its standard deviation.

Sensor	Penicillin Sensitivity Portable “Sensit Smart”	Penicillin Sensitivity Stationary “IM6ex”
1	104 ± 6 mV/dec	101 ± 6 mV/dec
2	101 ± 10 mV/dec	95 ± 9 mV/dec
3	101 ± 8 mV/dec	99 ± 8 mV/dec
Average	102 ± 2 mV/dec	98 ± 3 mV/dec

## Data Availability

The data presented in this study is available on request from the corresponding author.

## Supplementary Materials

The following supporting information can be downloaded at: https://www.mdpi.com/article/10.3390/s25092681/s1. Figure S1. Startup window; Figure S2. Setup window; Figure S3. Leakage current window; Figure S4. Capacitance-Voltage (*C*–*V*) window; Figure S5. Impedance spectrum window; Figure S6. Constant-Capacitance (ConCap) window.
