# Supplementary material for: Portable Measurement System for the Characterization of Capacitive Field-Effect Sensors
Source: Sensors (Basel). 2025 Apr 24;25(9):2681. doi: 10.3390/s25092681 (PMC12074279; doi:10.3390/s25092681)
Supplement: Supplementary file 1 [file sensors-25-02681-s001.zip › sensors-3594615-supplementary.pdf]

Supplementary material

Portable measurement system for the characterization of capacitive field-effect sensors

Tobias Karschuck [1,2], Stefan Schmidt [1,3], Stefan Achtsnicht [1], Joey Ser [1], Ismail Bouarich [1], Georges Aboutass [1], Arshak Poghossian [4], Patrick H. Wagner [2] and Michael J. Schöning [1,5,*]

[1] Institute of Nano- and Biotechnologies, FH Aachen, 52428 Jülich, Germany
[2] Laboratory for Soft Matter and Biophysics, KU Leuven, 3001 Leuven, Belgium
[3] Institute of Pharmaceutical Chemistry, Phillipps University of Marburg, 35037 Marburg
[4] MicroNanoBio, 40479 Düsseldorf, Germany
[5] Institute of Biological Information Processing (IBI-3), Forschungszentrum Jülich GmbH, 52425 Jülich, Germany
* Correspondence: schoening@fh-aachen.de

Graphical user interface

The graphical user interface was designed to be easy to navigate for the characterization of electrolyte-insulator-semiconductor capacitors (EISCAPs) using CustomTkinter, see reference [73] of the article. The startup window is shown in Figure S1. Blue buttons on the left side are used to select the setup menu (Setup) and display completed measurements (Leakage current, *C*–*V*, Impedance, ConCap). The mint switches next to the buttons can be used to select / unselect a measurement mode. The switch next to the "Setup" button is used to switch between a set working point capacitance and the automatic working point determination. The mint "Start" button starts a measurement and the red "Stop" button can be used to stop a running measurement. Below the "Stop" button is a progress bar, which indicates the completion state of the measurement. The currently running measurement mode is indicated by highlighting the respective button.

Figure S2 displays the "Setup" window, which is opened when the "Setup" button is pressed. Metadata concerning the sensor (wafer number of the sensor, sensor number, buffer composition and pH value) can be input into the entry fields. Additionally, the working point capacitance and measurement time for the constant-capacitance (ConCap) mode measurement can be set here. The default ConCap duration is set to 5 min (300 s). If no working point capacitance is set, it will be automatically determined at 60% of the maximum capacitance of the *C*–*V* curve. In that case, a ConCap measurement can only be performed after a preliminary *C*–*V* curve was collected.

Figure S3 displays the "Leakage current" window. After a measurement is completed, the measurement data is saved in the ".txt" format. When the "Leakage current" button is pressed, the leakage current files are plotted as ".png" images. Initially, the plot for the first performed measurement of the session is displayed. The navigation is handled by the buttons on the top of the figure: "Previous" and "Next" can be used to flip through the plots of the completed measurements. In between the two buttons, the number of the currently selected measurement is displayed in the dropdown menu, which can also be used to select a specific measurement. The program keeps track of already plotted files. Figures S4, S5 and S6 show the windows of the *C*–*V*, impedance and ConCap modes, respectively.

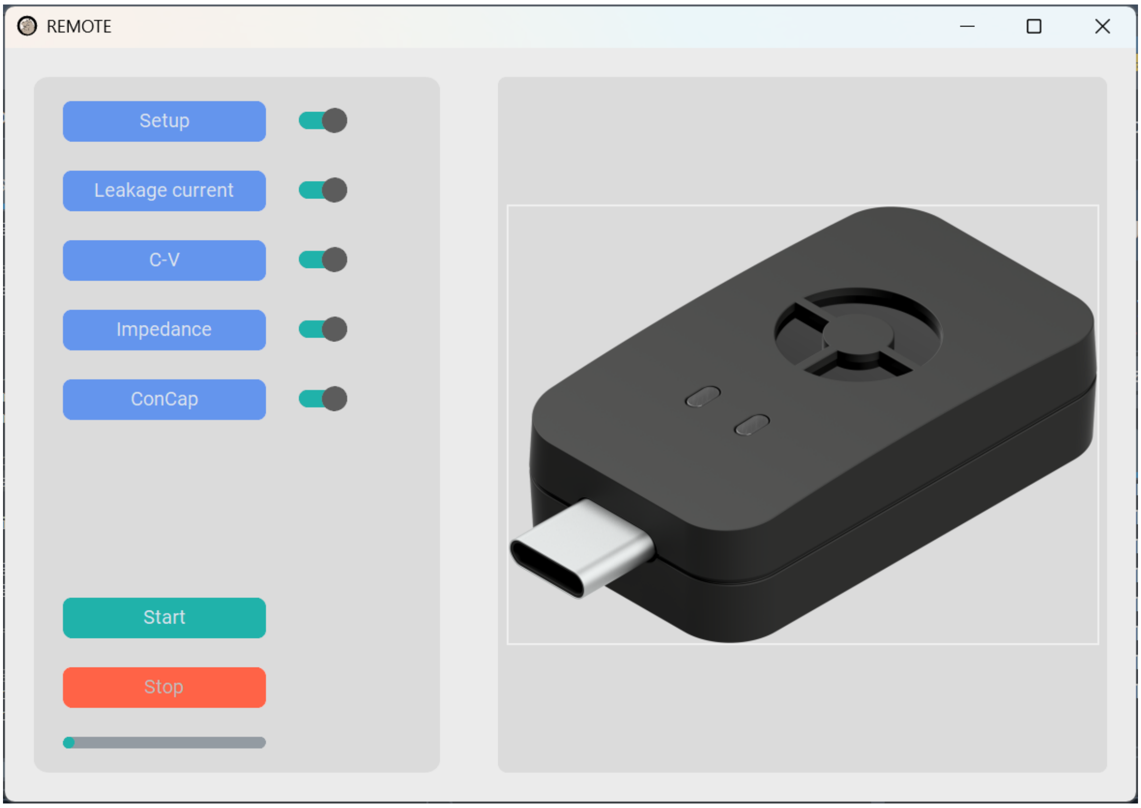

Figure S1. Startup window.

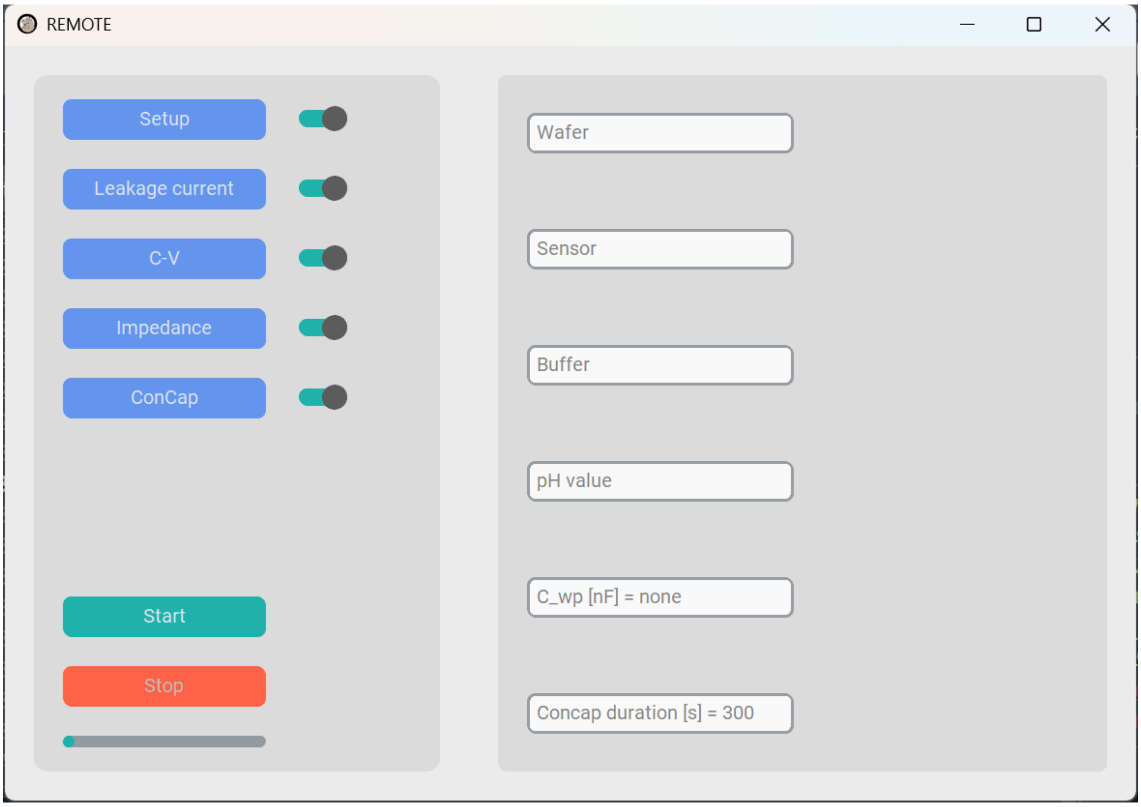

Figure S2. Setup window.

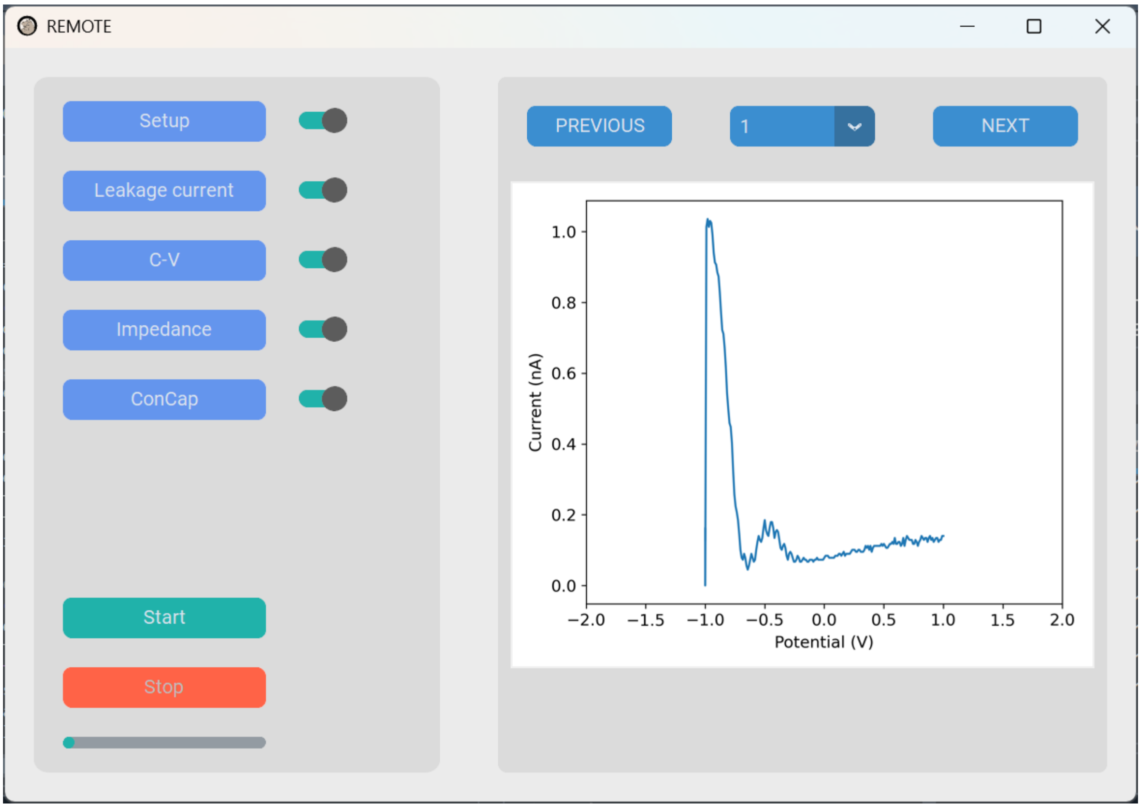

Figure S3. Leakage current window.

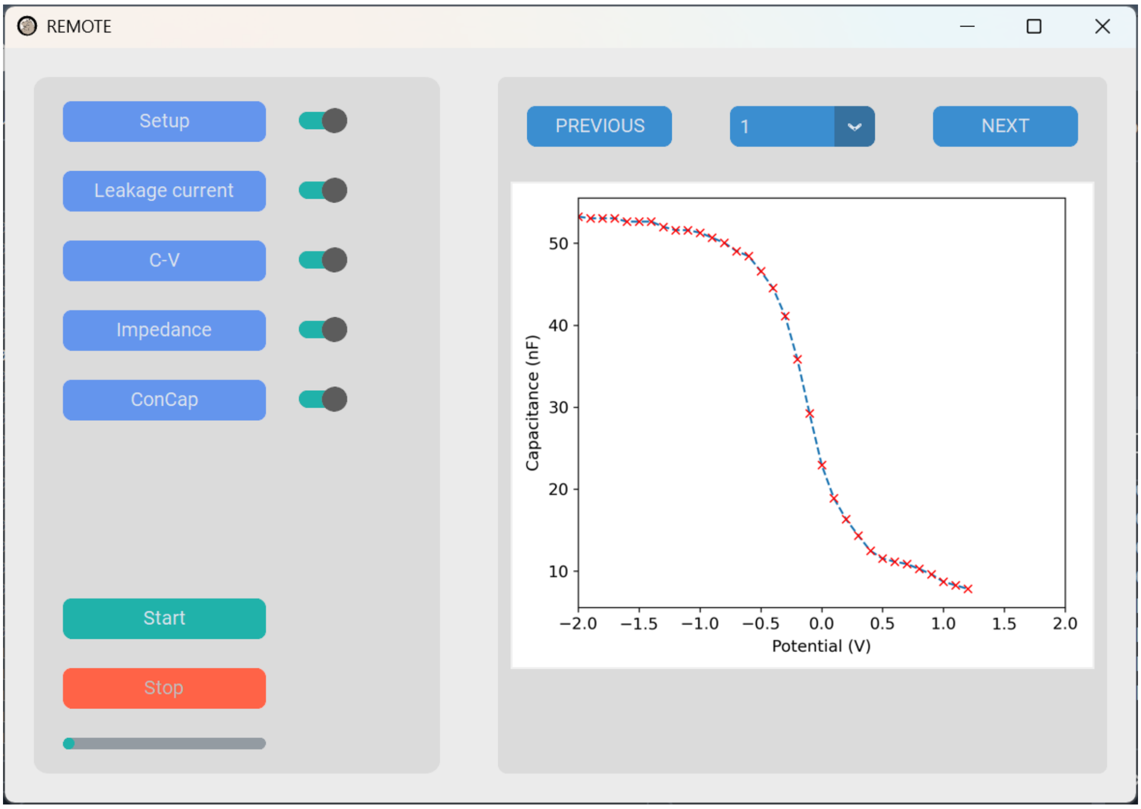

Figure S4. Capacitance-Voltage (*C*–*V*) window.

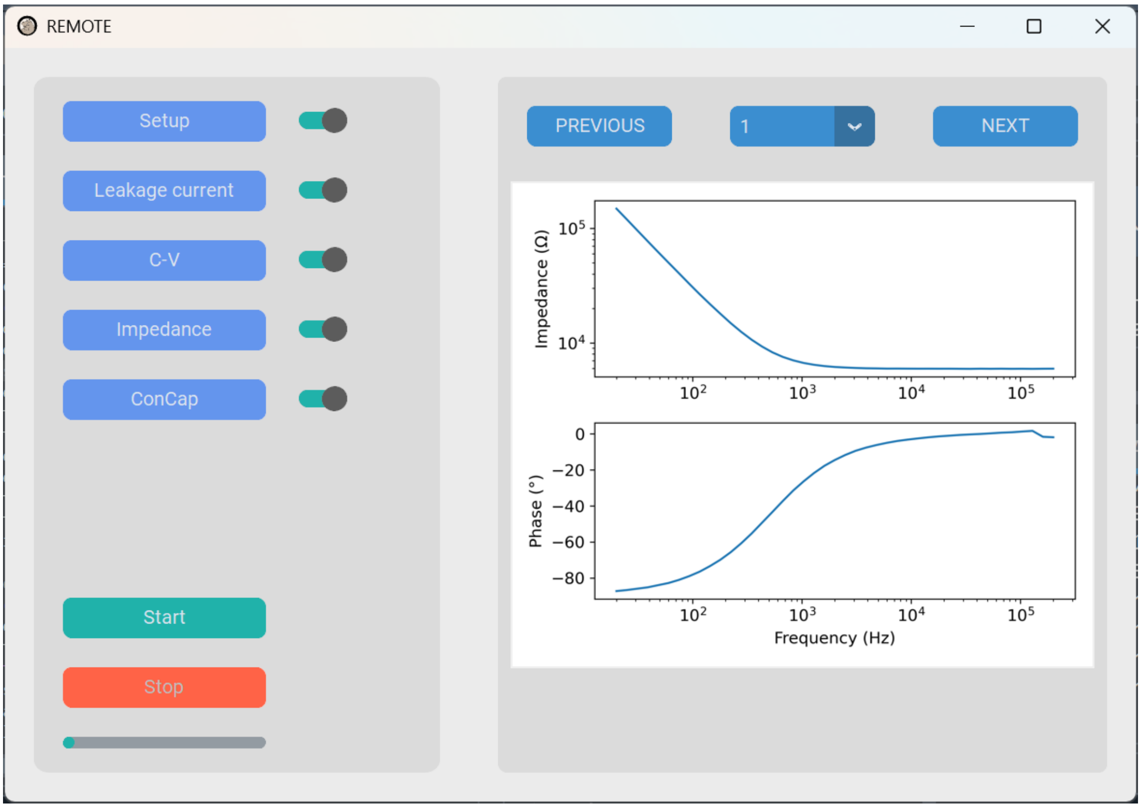

Figure S5. Impedance spectrum window.

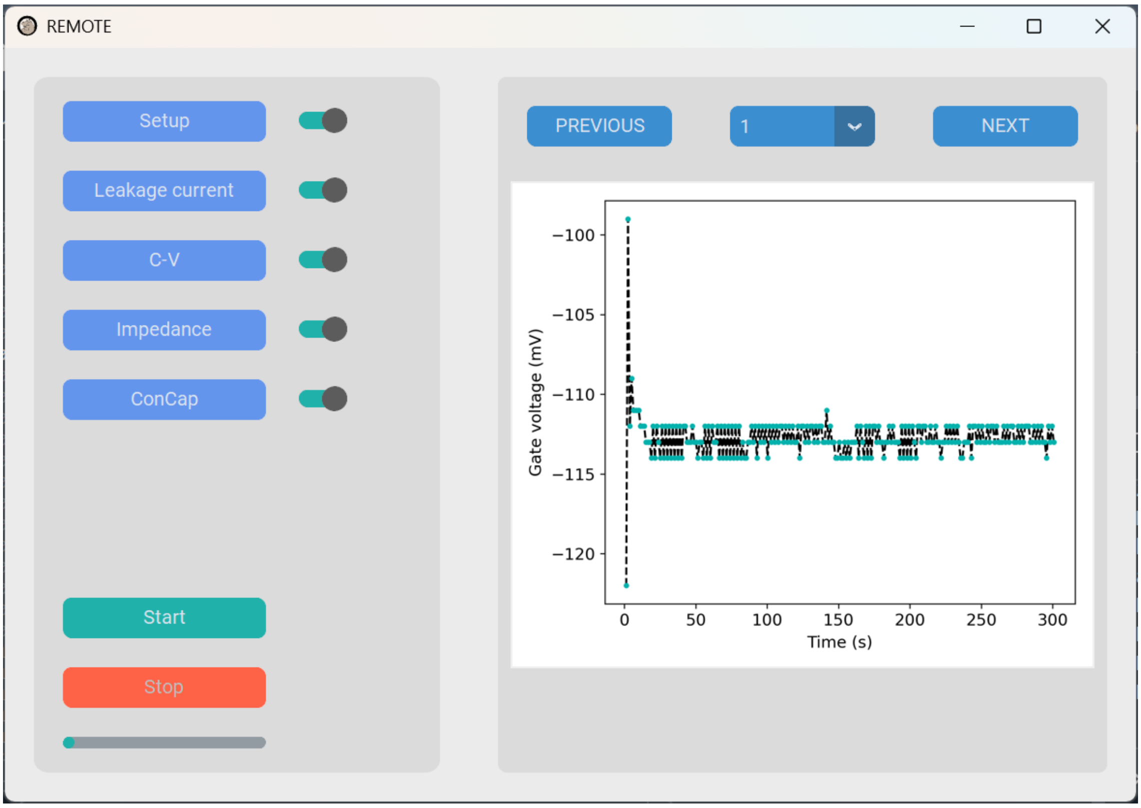

Figure S6. Constant-Capacitance (ConCap) window.
